# Exponential dosing to standardize myocardial perfusion image quality with rubidium-82 PET

**DOI:** 10.1007/s12350-023-03303-6

**Published:** 2023-05-31

**Authors:** Anahita Tavoosi, Ritika Khetarpal, R. Glenn Wells, Rob S. B. Beanlands, Robert A. deKemp

**Affiliations:** 1 Department of Medicine (Cardiology), University of Ottawa Heart Institute, 40 Ruskin Street, Ottawa, ON K1Y 4W7 Canada; 2 School of Interdisciplinary Science (Life Sciences), McMaster University, 1280 Main St W, Hamilton, ON L8S 4L8 Canada

**Keywords:** Positron emission tomography, coronary artery disease, myocardial perfusion imaging, image quality, patient centered imaging

## Abstract

**Background:**

^82^Rb PET is commonly performed using the same injected activity in all patients, resulting in lower image quality in larger patients. This study compared ^82^Rb dosing with exponential vs proportional functions of body weight on the standardization of myocardial perfusion image (MPI) quality.

**Methods:**

Two sequential cohorts of N = 60 patients were matched by patient weight. Rest and dipyridamole stress ^82^Rb PET was performed using 0.1 MBq·kg^−2^ exponential and 9 MBq·kg^−1^ proportional dosing. MPI scans were compared qualitatively with visual image quality scoring (IQS) and quantitatively using the myocardium-to-blood contrast-to-noise ratio (CNR) and blood background signal-to-noise ratio (SNR) as a function of body weight.

**Results:**

Average (min–max) patient body weight was 81 ± 18 kg (46–137 kg). Proportional dosing resulted in decreasing CNR, SNR, and visual IQS with increasing body weight (*P* < 0.05). Exponential dosing eliminated the weight-dependent decreases in these image quality metrics that were observed in the proportional dosing group.

**Conclusion:**

^82^Rb PET dosing as an exponential (squared) function of body weight produced consistent stress perfusion image quality over a wide range of patient weights. Dramatically lower doses can be used in lighter patients, with the equivalent population dose shifted toward the heavier patients to standardize diagnostic image quality.

**Supplementary Information:**

The online version contains supplementary material available at 10.1007/s12350-023-03303-6.

## Introduction

Myocardial perfusion imaging (MPI) with positron emission tomography (PET) provides high diagnostic accuracy compared to single photon tomography (SPECT) due mainly to higher sensitivity and accurate attenuation correction.^1–4^ We and others have demonstrated the prognostic value of rubidium-82 (^82^Rb) cardiac PET for risk-stratification in patients with coronary artery disease, particularly in those with obesity.^5–8^ Despite these advantages of ^82^Rb PET, image quality can still be affected by the patient’s body habitus as an increase in the body dimension leads to higher fractions of attenuated and scattered photons resulting in fewer recorded counts and increased image noise.^9^

Selecting an appropriate imaging protocol including administered activity appropriate for each patient’s body habitus is very important to standardize diagnostic image quality. Current SPECT imaging guidelines from the American Society of Nuclear Cardiology (ASNC) suggest “…an effort to tailor the administered activity to the patient’s habitus and imaging equipment should be made… [however] strong evidence supporting one particular weight-based dosing scheme does not exist.”^10,11^ Similarly for PET, the current ASNC perfusion imaging guidelines suggest that “Large patients may benefit from higher doses” but no specific recommendations are provided to ensure consistent image quality for ^82^Rb MPI.^12^

Image smoothing can help to reduce noise and improve image quality, but at the expense of lower spatial resolution.^9^ Alternatively, longer scanning times and/or weight-based tracer dosing have been proposed and are currently recommended as a solution to help standardize image quality in whole-body oncology PET imaging with F-18-fluorodeoxyglucose (^18^FDG).^13–15^ Historically, ^82^Rb PET imaging has been performed using a single constant dose for all patients^16^ due in part to limitations of early generator systems which were calibrated for dose delivery at a single activity value^17^ but this is known to result in lower count-density and corresponding lower image quality in larger patients. We have shown previously that this variation of image quality can be mitigated to some degree by the administration of activity in proportion to body weight (15) using a new generation ^82^Rb elution system.^18^ Contrary to ^18^FDG PET imaging however, longer scan times can not be used to improve ^82^Rb image quality in these patients due to the ultra-short half-life of 75 seconds.

The European Association of Nuclear Medicine (EANM) guidelines for PET MPI currently recommends weight-based tracer dosing for ^82^Rb imaging in 3D-mode at 10 MBq·kg^−1^ (with a minimum dose of 740 MBq and maximum of 1480 MBq),^19^ whereas the ASNC PET MPI guidelines still accept the use of a single constant dose of ^82^Rb ranging from 740 to 1110 MBq depending on the PET-CT device sensitivity.^10^ The common lower limit of 740 MBq may not allow adequate dose reduction in very small patients, whereas the upper limit of 1110 to 1480 MBq may not allow adequate image quality in the largest patients.

Our center has, for several years, used weight-based dosing as a proportional function of patient weight (9-10 MBq·kg^−1^) to reduce variations of image quality depending on body habitus, and to reduce detector saturation during the tracer first-pass for accurate blood flow quantification.^1,20^ Despite this approach, larger patients still appear to suffer from reduced ^82^Rb PET image quality which is not aligned with the recommended principles of patient-centered imaging.^21,22^ Therefore, the aim of this study was to investigate whether ^82^Rb dosing as an exponential (squared) function of weight may help to standardize PET MPI quality across a wide range of patient body sizes, following a similar protocol validated previously for whole-body ^18^FDG PET.^23,24^

## Methods

### Study design

**Figure 1:**
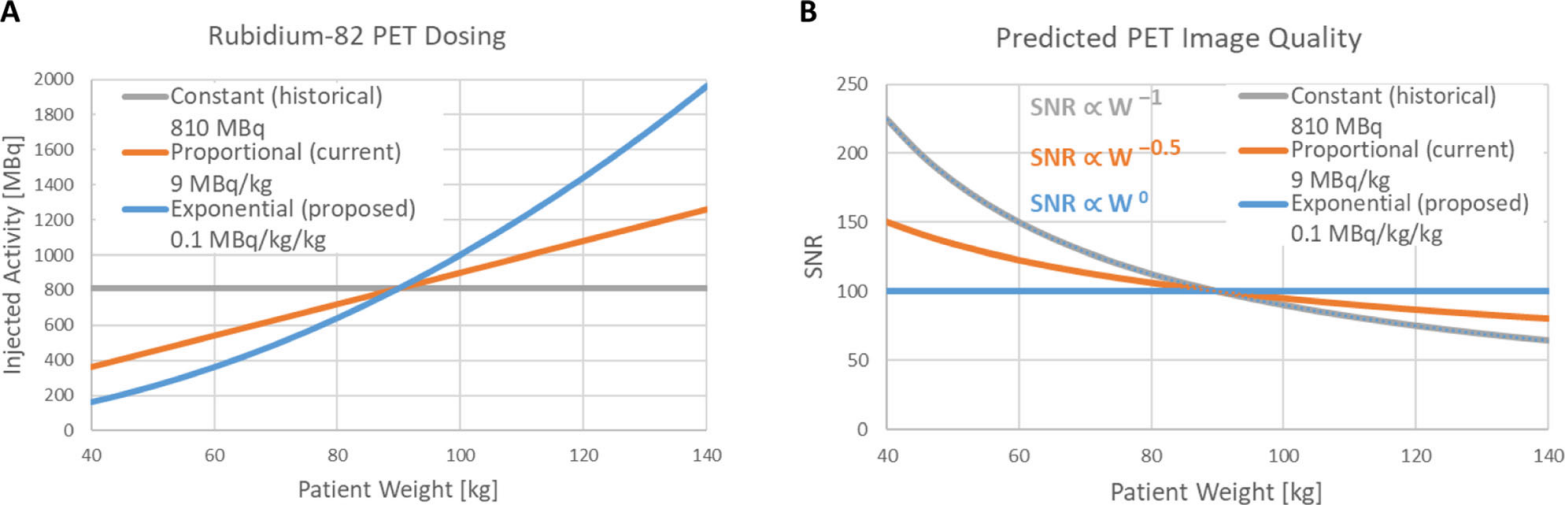
^82^Rb PET dosing protocols as a function of patient body weight. (A) Constant, proportional and exponential dosing curves intersect at a common injected activity (810 MBq) and average patient body weight (90 kg). (B) Predicted changes in signal-to-noise ratio (SNR) as a function of patient weight for 3 different dosing methods (scaled to 100% at 90 kg) based on previous ^18^FDG PET studies by de Groot^23^ and Koopman^24^

This was an interrupted time series cohort comparison study performed as part of the clinical quality improvement (CQI) program in the Cardiac Imaging department at the University of Ottawa Heart Institute, therefore the requirement for informed patient consent was waived by the Ottawa Health Science Network Research Ethics Board. An exponential dosing protocol was designed to increase the ^82^Rb activity as a squared function of body weight, while maintaining the same injected activity as the previous proportional dosing function for patients with our historical population average weight of 90 kg, as illustrated in Figure 1A.

PET image quality is determined by count statistics which follow a Poisson distribution. As a first-order approximation, with statistical iterative reconstruction methods the local image variance is proportional to the mean activity concentration (or the total number of radioactive decays recorded), and therefore the local image signal-to-noise ratio (SNR) should be proportional to the square-root of the local activity concentration (or total injected activity) and imaging time, i.e., ${\text{SNR}}= \sqrt{At} \times k$, where the parameter *k* is a constant specific to the PET scanner, image reconstruction protocol and target organ.

The standard relationship above was extended by de Groot to include patient weight effects observed empirically in ^18^FDG PET studies of the liver^20^ according to Eq. 1.

1 $$ {\text{SNR}}_{{{\text{Target}}}} = \sqrt {At} \times k \times {\text{Weight}}^{\beta } $$

For ^82^Rb PET, the scan time (*t*) is essentially fixed, therefore SNR is determined solely by the injected activity (*A*). In the case of constant injected activity, SNR_LIVER_ has been shown to decrease as an exponential function of weight (*β* =  − 1) as illustrated in Figure 1B.^23^ With proportional dosing (*A* ∝ Weight) image SNR still decreases with patient weight, but with a lesser dependence (i.e., *β* =  − 0.5). Finally, if activity is administered as a squared function of weight (*A* = *ε* × Weight^2^) and scan time is fixed, then SNR is expected to remain constant (*β* = 0) across different patient weights as derived in Eq. 2 and illustrated in Figure 1B.

2 $$ {\text{SNR}}_{{{\text{Constant}}}} = \sqrt {\varepsilon \times {\text{Weight}}^{2} } \times k \times {\text{Weight}}^{ - 1} = \sqrt \varepsilon \times k $$

where the dosing parameter ε is site-dependent and can be adjusted to obtain the desired SNR_Constant_ value in the target organ using a particular scanner and image reconstruction protocol. In this study a value of *ε* = 0.1 MBq·kg^−2^ was selected to maintain the same injected activity (810 MBq) in our historical average patient weight of 90 kg.

### Patient population

**Figure 2:**
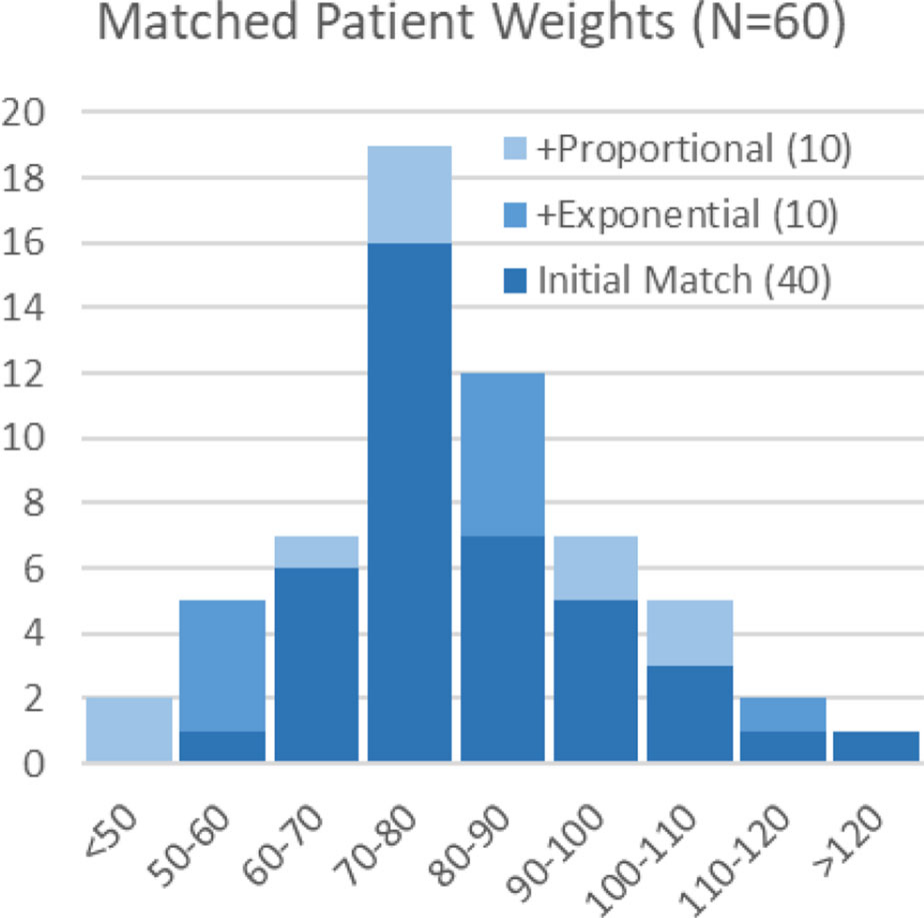
Patient weight distributions in the exponential and proportional dosing cohorts were matched prospectively

A control group of 50 consecutive patients was identified initially who underwent clinically indicated ^82^Rb MPI imaging with *proportional* dosing (9 MBq·kg^−1^) during a 2-week period in November 2020. Following a short transition period, an additional 50 consecutive patients who underwent clinically indicated ^82^Rb myocardial perfusion imaging (MPI) with the *exponential* dosing protocol (0.1 MBq·kg^−2^) were identified during a 1-week period in January 2021. The distribution of patient weights was compared between cohorts in 10 kg intervals as shown in Figure 2. In those intervals with unequal numbers, subsequent consecutive patients in each cohort (N = 10) were added to obtain a final matched weight distribution consisting of N = 60 patients in both groups.

### ^82^Rb PET imaging

Both proportional and exponential cohort scans were acquired on a Biograph Vision600 PET-CT scanner (Siemens Healthcare, Hoffman Estates, IL) following our standard clinical protocols.^25^ Briefly, a single low-dose CT scan was performed at normal end-expiration for attenuation correction of the rest and stress PET scans. Dynamic PET imaging was performed at rest and again during dipyridamole stress (0.14 mg·kg^−1^·min^−1^ × 4 min). For both scans, a 30-seconds square-wave injection of Rubidium Rb 82 Chloride injection (RUBY-FILL™, Jubilant Radiopharma, QC) was administered followed by a 20 mL saline-push.^25^ Ungated static images were reconstructed from 2 to 8 minutes, ECG-gated images (8 bins per cycle) from 1 1/2 to 8 minutes following tracer injection to maximize count statistics following the blood clearance phase. The vendor iterative OSEM reconstruction method was used including time-of-flight with 5 subsets, 4 iterations, 128 matrix size with 4 × 4 × 3 mm^3^ voxels and 6 mm Gaussian post-filtering.

### Image quality analysis

Visual image quality was determined for the stress ECG-gated series independently by two experienced physicians (AT, RSB) blinded to the study cohorts and to each other’s results. Image quality scores in the heart (IQS_HEART_) were assessed using a 5 point-scale (poor, fair, good, very good, excellent) based on the visual interpretation of heart-to-blood contrast and background noise as shown in Supplemental Figure S1. Intermediate (1/2 point) scores were also allowed resulting in 9 discrete scoring levels. Reliability between operators was assessed using Bland–Altman analysis, and the averaged scores were used in the final analysis.

**Figure 3:**
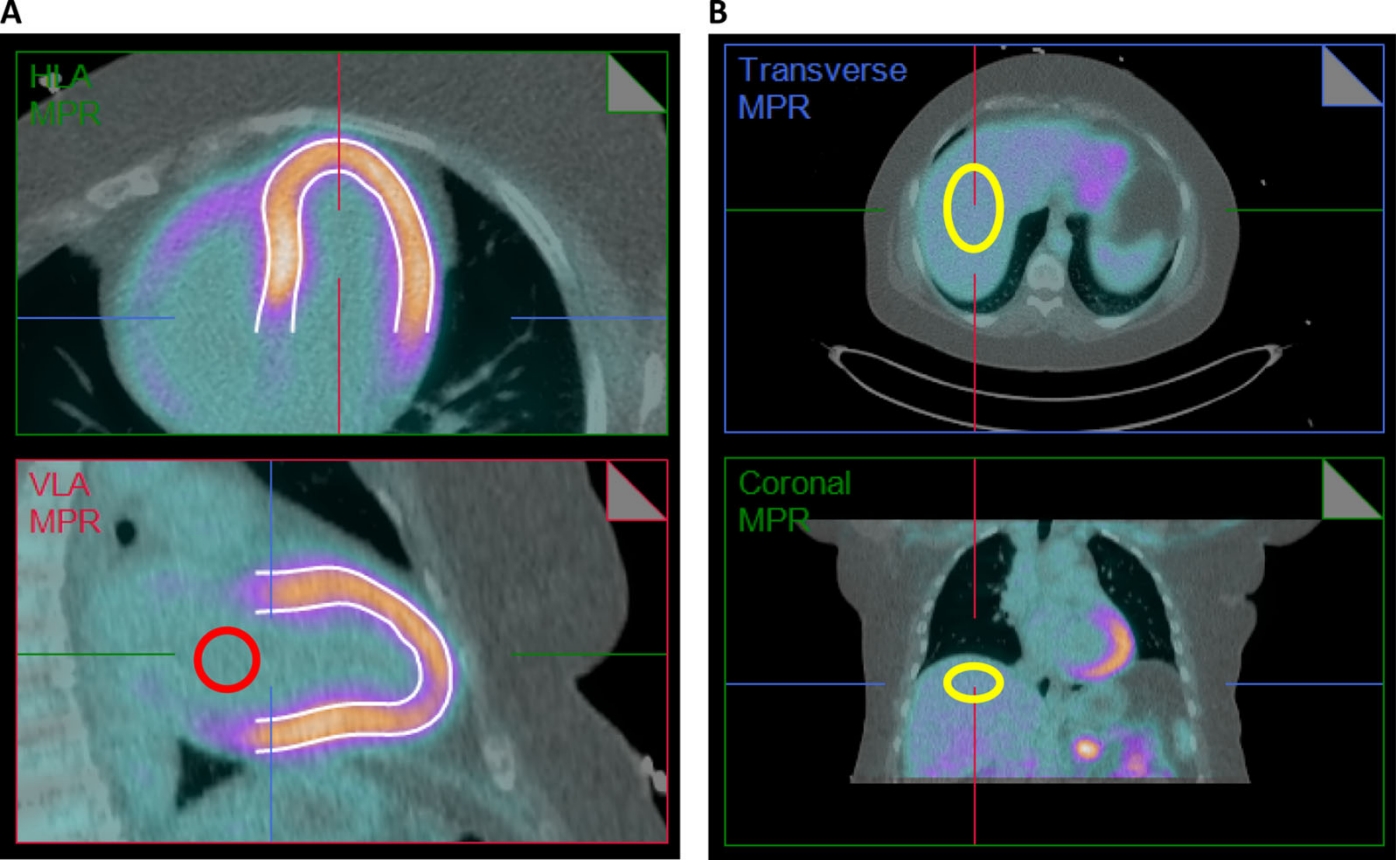
Regions-of-interest drawn in the heart (A) and liver (B) for measurement of CNR and SNR. LV_MAX_ was taken within the three-dimensional region of the myocardial wall (white) identified automatically by the Corridor-4DM software. Blood mean and standard deviation were taken in a single-slice region drawn manually in the left atrial cavity (red) on a vertical long axis (VLA) image. Liver mean and standard deviation were taken in an ellipsoid volume-of-interest drawn manually near the diaphragm (yellow)

Quantitative stress image analysis was performed using Corridor-4DM software v2018 (INVIA Medical Solutions, Ann Arbor, MI). Myocardium signal was measured as the maximum LV activity (LV_MAX_) to avoid the effects of tracer uptake defects due to regional coronary disease. The blood background signal and noise were measured as the left atrium cavity mean and standard deviation (Blood_MEAN_ and Blood_SD_) in a blood region drawn manually as shown in Figure 3. Contrast-to-noise in the heart (CNR_HEART_) = (LV_MAX_ − Blood_MEAN_)/Blood_SD_ and SNR_BLOOD_ = Blood_MEAN_/Blood_SD_ were calculated for both the ungated (static) and ECG-gated (end-diastolic) stress PET images. Measurements of LV_SD_ were not available in the 4DM software therefore a myocardial-specific SNR was not computed. To ensure reliability of these semi-automated measurements, two operators performed the heart CNR and blood SNR analyses (AT, RDK), blinded to the study cohorts and to the results of the other operator. These values were averaged between operators and used in the analyses of weight-based and dosing-based effects. To enable direct comparison of ^82^Rb to the ^18^FDG exponential dosing results of de Groot et al. image quality was also measured in the liver.^23^ SNR was measured as the mean divided by the standard deviation (SD) of activity in a large volume of interest (VOI) drawn in an area of uniform uptake in the liver, i.e., SNR_LIVER_ = Liver_MEAN_/Liver_SD_ as shown in Figure 3.

To characterize the dependence of image quality on patient body weight, the visual IQS_HEART_, and quantitative CNR_HEART_, SNR_BLOOD_, and SNR_LIVER_ values were plotted against patient weight, and the data fit to exponential power functions as shown in Eq. 3.

3 $$ {\text{IQS}} \left| { {\text{CNR}} } \right| {\text{SNR}} = \alpha \times {\text{Weight}}^{\beta } $$

where the parameter $\alpha =\sqrt{\varepsilon }\times k$ from Eq. 2, and the exponent β indicates whether image quality is increasing (*β* > 0), decreasing (*β* < 0) or is constant (*β* = 0) as a function of patient weight.

### Statistical analysis

Measurements of IQS, SNR, and CNR were compared between operators using Bland–Altman analysis. The weight-dependence of image quality on body weight (*β* coefficients) were compared between the exponential and proportional dosing groups using 95% confidence intervals. Variances were compared using non-parametric Levene’s tests. Mean values were compared using paired Student *t*-tests, and median values using Mann–Whitney *U* tests. *P* < 0.05 was considered statistically significant. Statistical analysis was performed using Excel 2019 with Real Statistics 8.1.

## Results

Patient demographics are shown in Table 1. The proportional and exponential dosing cohorts had similar clinical characteristics, including patient weights (80.9 ± 18.2 kg and 81.0 ± 17.7 kg; *P* = 0.96) as expected based on the prospective cohort matching (Figure 2). The median injected activity was 12% lower using exponential vs proportional dosing (*P* = 0.04), as the median weight in our experimental cohort (80 kg) was slightly lower than the historical value of 90 kg used to design the exponential dosing protocol. The min–max range was substantially wider (211–1850 vs 433–1362 MBq) as expected using exponential vs proportional dosing.

**Table 1:** Patient demographics

Description	Proportional dosing (N = 60)	Exponential dosing (N = 60)	*P*-value
Age (years)	65 ± 14	69 ± 11	0.09
Female sex	27 (45%)	28 (47%)	0.86
Weight (kg)	81 ± 18	81 ± 18	0.96
Body Mass Index (kg·m^−2^)	29 ± 7.5	29 ± 6.2	0.92
Coronary risk factors			
Hypertension	39 (65%)	41 (68%)	0.70
Dyslipidemia	43 (72%)	45 (75%)	0.68
Family history	28 (47%)	26 (43%)	0.71
Smoking (current or past)	35 (58%)	36 (60%)	0.85
Diabetes (type I or II)	15 (25%)	14 (23%)	0.83
Angina symptoms			
None	35 (58%)	28 (47%)	0.20
Typical	8 (13%)	10 (17%)	0.61
Atypical	5 (8%)	10 (17%)	0.17
Non-anginal	12 (20%)	12 (20%)	1.00
Cardiac history			
Previous myocardial infarction	10 (17%)	19 (32%)	0.06
Previous percutaneous intervention	11 (18%)	12 (20%)	0.82
Previous coronary bypass grafting	3 (5%)	6 (10%)	0.30

Values are mean ± standard deviation or N (%)

No significant differences between dosing cohorts

**Figure 4:**
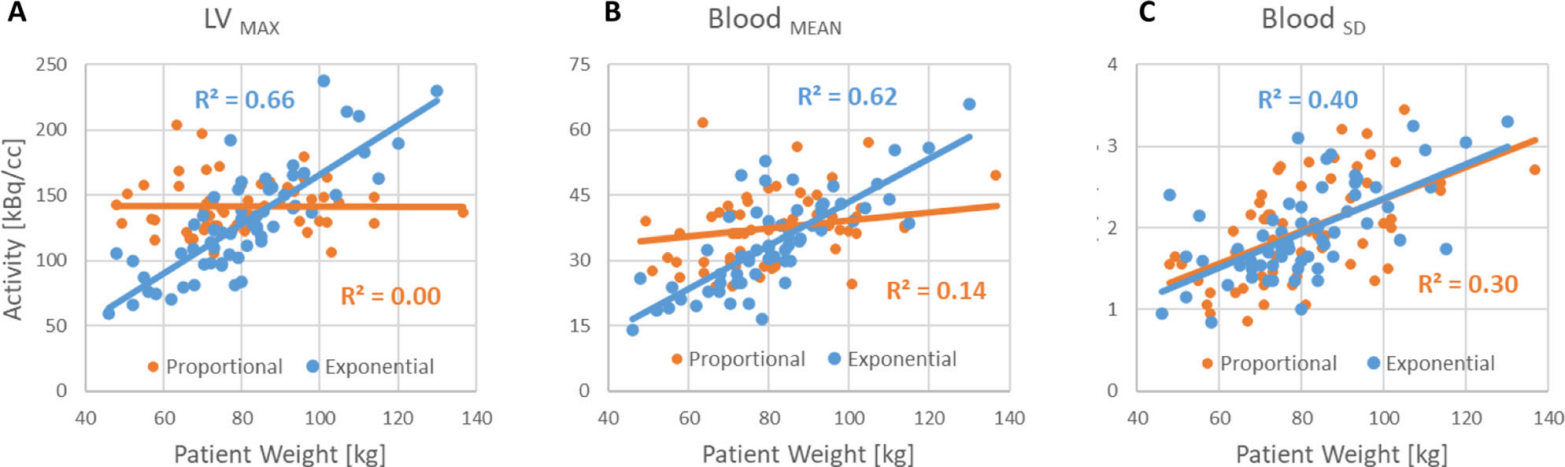
^82^Rb PET activity values on ECG-gated imaging with proportional and exponential dosing. LV_MAX_ (A) values are constant with proportional dosing (orange) but increase linearly by weight with exponential dosing (blue). (C) Blood_SD_ activity remains very similar between dosing protocols

With proportional dosing the measured activity values in the LV myocardium and blood were relatively constant, whereas with exponential dosing they both increased linearly with patient body weight (Figure 4A, B). Background noise (Blood_SD_) in both cohorts increased linearly with body weight and was unchanged between dosing protocols (Figure 4C).

For the measurements of cardiac IQS, CNR, and SNR, the inter-operator agreement was excellent with mean differences ≤ 5% (details in Supplemental Table S1). The average values of IQS, CNR, and SNR are shown for both dosing cohorts in Table 2. In the exponential dosing cohort, there was an average decrease of − 8.5% across all image quality metrics, consistent with the lower average injected activity as noted earlier. More importantly, there was 40% decreased variability of both the static and gated CNR_HEART_ values in the exponential dosing cohort (*P* < 0.001) demonstrating significantly improved consistency of image quality compared to proportional dosing.

**Table 2:** ^82^Rb PET image quality measurements

Image quality	Proportional	Exponential
IQS_HEART_		
Gated	3.3 ± 0.5	3.1 ± 0.6
CNR_HEART_		
Static	117 ± ***45***	95 ± 27*
Gated	61 ± ***23***	51 ± 14*
SNR_BLOOD_		
Static	30 ± 8	27 ± 6
Gated	21 ± 5	18 ± 5
SNR_LIVER_		
Static	19 ± 3.8	19 ± 4.2
Gated	16 ± 3.5	15 ± 3.6

Values are mean ± standard deviation

*IQS*, Image Quality Score, *SNR*, Signal-to-Noise Ratio, *CNR*, Contrast-to-Noise Ratio

*P < 0.001 lower variance versus proportional dosing cohort

**Figure 5:**
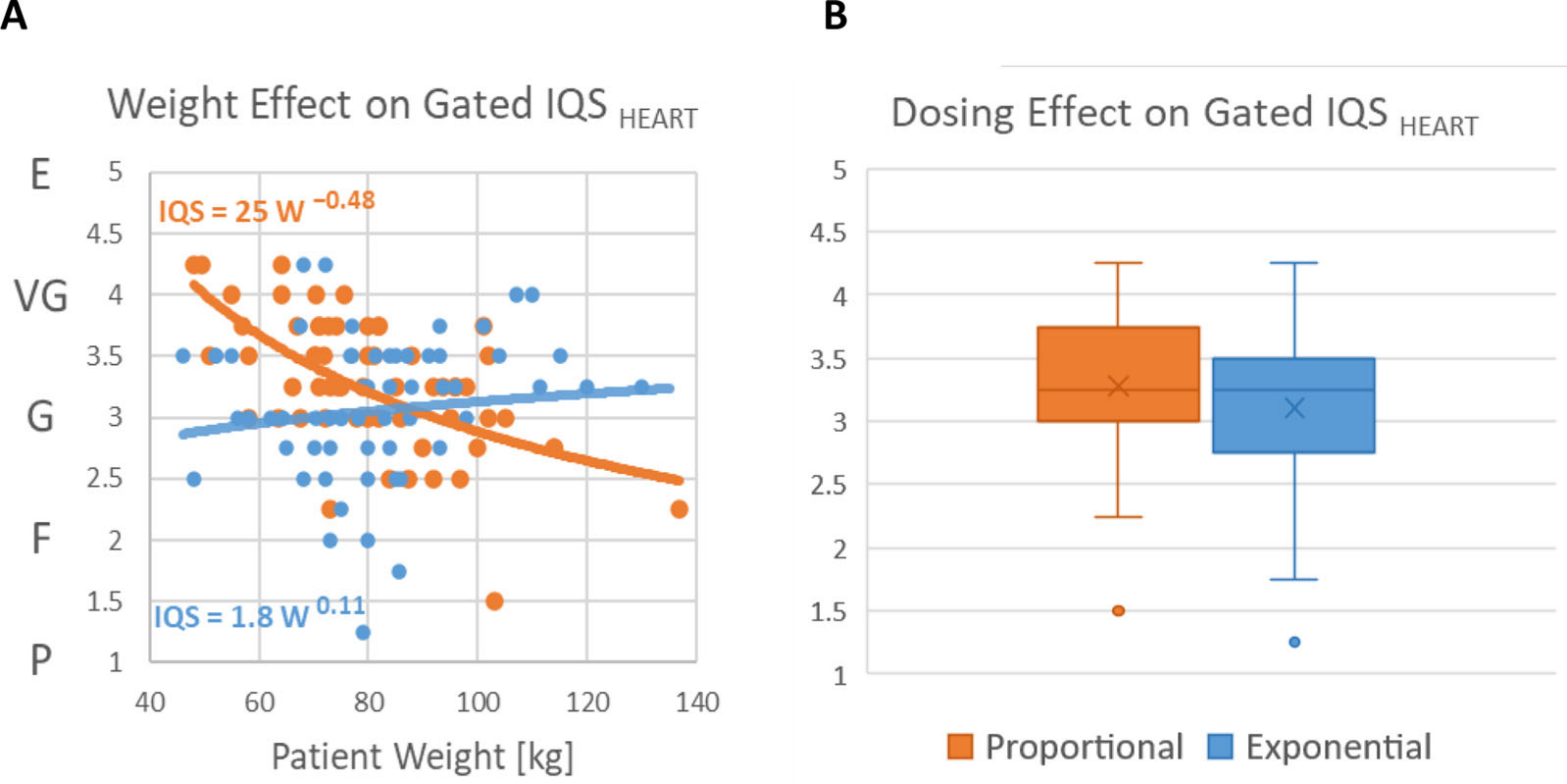
^82^Rb PET visual image quality score (IQS_HEART_) was assessed on a 5-point scale (Excellent, Very Good, Good, Fair, Poor) which *decreased* by weight (A) in the proportional dosing group (orange) but was *constant* in the exponential dosing group (blue). There was no difference in the median ECG-gated image quality score (B) between dosing cohorts (P = 0.11). Lines of best-fit are *IQS* ∝ *Weight*^β^

**Figure 6:**
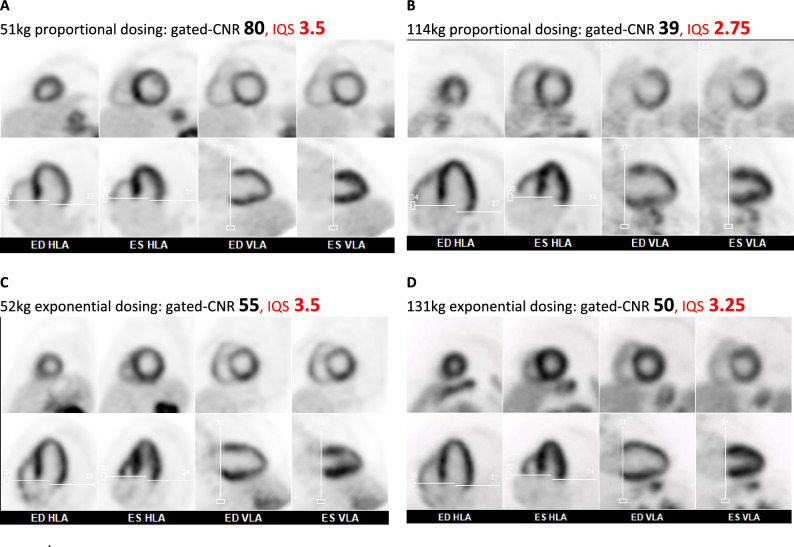
^82^Rb PET static-ungated SA (top) and ECG-gated HLA & VLA (bottom) images acquired with proportional (A,B) and exponential (C,D) dosing. Proportional dosing resulted in visibly lower image quality in the large (B) vs small (A) patient (CNR = 39 vs 80). With exponential dosing the image quality was very similar between the large (D) and small (C) patient (CNR = 50 vs 55), and much improved vs the large patient with proportional dosing (B)

Improved consistency was confirmed with the visual image quality scores (Figure 5) in the exponential dosing cohort, which showed no significant dependence on body weight (*β* = 0.11; *P* = 0.38). This was in contrast to the proportional dosing group which showed a significant decrease in image quality (*β* =  − 0.48; *P* < 0.001) that was very similar to the value predicted by Eq. 1 and shown in Figure 1B (*β* =  − 0.5). Interestingly, the crossing point of equivalent IQS_HEART_ values in both cohorts was close to 90 kg, further demonstrating validity of the noise model and dosing methods as described in the study design. Higher body weight was observed in the patients with lower IQS in the proportional dosing cohort (*P* < 0.001) but with not exponential dosing (*P* = 0.82) where the distribution of weights was uniform across different visual IQS values (Supplemental Figure S2). The changes in visual image quality between dosing methods can be seen in the patient examples shown in Figure 6 and Supplemental Figure S3.

**Figure 7:**
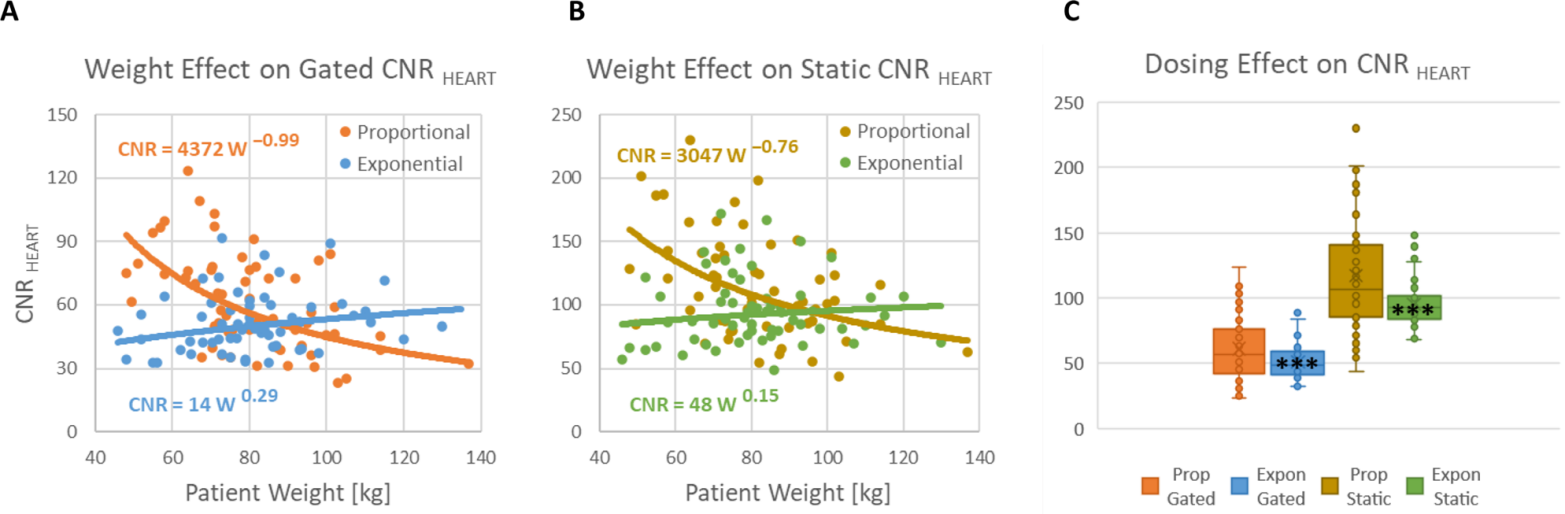
^82^Rb PET contrast-to-noise ratio (CNR_HEART_) *decreases* with increasing patient body weight in the proportional dosing cohort but not in the exponential dosing cohort for both ECG-gated (A) and ungated static (B) images. Box-plots of CNR_HEART_ in (C) show there was a highly significant effect of exponential dosing to reduce the variability in image quality (CNR_HEART_) among patients for both static and gated reconstructions (***P < 0.001 lower cohort variance versus proportional dosing). Lines of best-fit are *CNR* ∝ *Weight*^β^

The quantitative CNR_HEART_ values shown in Figure 7 demonstrated even more pronounced effects compared to the visual IQS_HEART_ scores. Both the ECG-gated and static images had better consistency of image quality in the exponential vs proportional dosing group (Figure 7A, B). Proportional dosing resulted in significantly decreased CNR_HEART_ with increasing weight (*β* =  − 0.99 and − 0.76, both *P* < 0.001), whereas there was no significant weight effect in the exponential dosing cohort (*β* = 0.29 and 0.08, both *P* > 0.05). The corresponding effects of dosing protocol on SNR_HEART_ and SNR_LIVER_ were also very similar, as shown in the Supplemental Figures S4 and S5.

The β coefficients summarizing the weight-dependence of all the image quality metrics are shown in Table 3. In the proportional dosing cohort, the average coefficient was (*β* =  − 0.56) confirming the negative effect of patient weight on image quality that was predicted in Figure 1B. In the exponential dosing cohort, the average coefficient was (*β* = 0.19) suggesting a possible small effect to actually increase quality in the gated and static images of the larger patients. This suggests that an exponential dosing coefficient slightly less than the squared function that we evaluated (exponent < 2) may have been sufficient to remove the weight-dependence of image quality. On the other hand, the squared function did produce very consistent results between visual IQS and quantitative CNR_HEART_ which were both based on the combined evaluation of myocardium to blood contrast and background noise.

**Table 3:** Weight-dependence of ^82^Rb PET image quality

*β* Coefficients	Proportional dosing	Exponential dosing	Exponential –Proportional
Gated IQS_HEART_	− 0.48*	+ 0.11	+ 0.59
Static CNR_HEART_	− 0.76*	+ 0.15	+ 0.91
Gated CNR_HEART_	− 0.99*	+ 0.29	+ 1.28
Static SNR_BLOOD_	− 0.28	+ 0.24	+ 0.52
Gated SNR_BLOOD_	− 0.46*	+ 0.35*	+ 0.81
AVERAGE_HEART_	** − 0.59**	** + 0.23**	** + 0.82**
Static SNR_LIVER_	− 0.39*	+ 0.01	+ 0.40
Gated SNR_LIVER_	− 0.56*	− 0.02	+ 0.54
AVERAGE_LIVER_	** − 0.48**	** − 0.01**	** + 0.47**

*P < 0.05 compared to zero

## Discussion

To our knowledge, this is the first report of a patient-centered approach using exponential dosing to standardize image quality for ^82^Rb PET perfusion imaging. In the control group, when ^82^Rb activity was administered in proportion to patient weight (9 MBq·kg^−1^) image quality was observed to decrease significantly with increasing body weight (*β* values < 0). For each 10 kg increase in patient weight, the ECG-gated CNR decreased by approximately 10%. This is equivalent to 50% reduction in CNR when the patient weight is doubled from 50 kg (110 lbs) to 100 kg (220 lbs), similar to the reduction shown in the patient examples of Figure 6A and B. Conversely, in the experimental group (Figure 6C and D) using exponential dosing (0.1 MBq·kg^−2^) the image quality was more consistent (*β* values ≈ 0) with less than 10% variation on average across a wide range of patient weights ranging from approximately 50 to 120 kg. The biggest changes in activity occurred at the extremes of patient weight, essentially redistributing the population dose from the smaller to the larger patients as needed to standardize image quality.

### Comparison to Guidelines and Previous Studies

The current ASNC guidelines advise either a constant dose for all patients or a proportional weight-based dose of ^82^Rb for PET perfusion studies,^12^ both of which have the limitation of producing lower quality images in obese patients. In the field of oncology PET, de Groot et al. found that ^18^FDG activity administered as a squared function of patient weight provided whole-body PET images of consistent quality, i.e., liver SNR no longer varied with patient weight.^23^ This exponential relation between ^18^FDG dose and body weight was also verified by Koopman^24^ for general implementation and independently by Musarudin et al.^26^ to provide constant liver image quality on a BGO PET-CT scanner. As a result of these studies, exponential or ‘quadratic’ dosing is now recommended for ^18^FDG PET-CT imaging in the most recent EANM procedure guidelines for tumor imaging.^15^ In the present study, the effects of exponential vs proportional ^82^Rb PET dosing on liver SNR were consistent with these previous studies of whole-body ^18^FDG PET.^13,23,24,26,27^ The de Groot model of image quality shown in Eq. 1 predicts that SNR_LIVER_ will decrease inversely as the square of patient weight (*β* =  − 0.5) which is consistent with the mean value of − 0.48 observed in our control cohort (Table 3). This weight-dependence was effectively eliminated in the exponential dosing cohort with an average *β* < 0.01, reproducing the results demonstrated previously using ^18^FDG PET.

The effects of proportional dosing to produce constant LV_MAX_ activity values in the heart (Figure 4) are partially consistent with results presented in the recent ^82^Rb PET study by van Dijk et al. who reported that the number of recorded ‘net’ coincidences (prompts–randoms) was constant over a wide range of patient weights.^28^ However, unlike this previous study which found no differences in body weight among the different categories of visual image quality with proportional dosing, the present study demonstrated statistically significant decreases in image quality (assessed visually and quantitatively) as a function of body weight, consistent with the model that was developed and validated previously for ^18^FDG whole-body PET.^20,24^ The pattern of decreasing image quality (despite constant tissue activity and ‘net’ coincidence counts) is likely due to the degrading effects of tissue attenuation on image quality. Our results suggest that the increasing noise effects of PET attenuation are approximately linear with patient weight, and these can be corrected with the exponential dosing protocol, to produce organ activity values that increase linearly with weight. It is surprising to us that van Dijk et al.^28^ did not find a significant weight-effect of image quality using their proportional dosing protocol, however there are some methodological factors in their study which may have contributed: 1. Indirect evaluation of the weight distribution of patients across different image quality scores, 2. PMT-based PET scanner with lower sensitivity and resolution, 3. Visual evaluation of static images only where noise effects are less apparent vs ECG-gated, 4. Use of a ^82^Rb generator system designed for single (constant) dose imaging.^29^

In contrast to our findings of improved standardization using exponential dosing with rubidium PET, a previous study with technetium SPECT perfusion imaging found that image noise in the LV myocardium could be standardized using the product of injected activity and scan-time adjusted as a proportional function of patient weight.^30^ While image quality using both these modalities is affected by the Poisson distribution of counting statistics, the noise effects and correction methods for the physical effects of scatter and attenuation (as well as random and prompt-gamma coincidences in PET) are quite different, which may explain the different results in SPECT vs PET.

Our results have important implications for pediatric imaging studies such as Kawasaki Disease where PET imaging has been used to guide clinical management.^31^ In children, the effective dose constant (radiation risk) is typically higher per unit activity injected (e.g., 4.9 vs 1.1 mSv·GBq^−1^ in a 5-year-old vs adult patient) reflecting the higher organ activity concentrations and smaller distances between organs.^32^ Our results suggest that the injected activity (and radiation effective dose) can be substantially reduced in the smallest patients while still maintaining diagnostic image quality.

### Clinical implementation

The exponential dosing protocol for ^82^Rb was easy to implement clinically by the PET technologists as a simple calculation, i.e., activity = weight (kg) × weight (kg)/10. For example, an 85 kg patient would be prescribed the ^82^Rb dose of 85 × 8.5 = 722.5 MBq (19.5 mCi). Patients of 149 kg would be given the maximum dose of 2220 MBq (60 mCi) listed in the U.S. package insert^25^ or 3700 MBq (100 mCi) for a 193 kg (425 lbs) patient as listed in the Canadian monograph.^33^ The activity available from the ^82^Rb generator decreases over time according to the half-life of the parent ^82^Sr, from 3700 MBq on day 0 to 700 MBq on day 60. Therefore, to implement exponential ^82^Rb dosing in practice, patient scheduling needs to be adjusted accordingly, with maximum patient weights up to 193 kg on day 0 and up to 84 kg on day 60.

The present study results may be adapted to other PET perfusion imaging protocols, taking into account the differences in tracer retention fraction, isotope half-life, scan-time, and PET scanner sensitivity. ^82^Rb has approximately 30% tracer retention in the heart at a peak stress blood flow value of 3 mL·min^−1^·g^−1^, whereas other PET tracers such as ^13^N-ammonia or ^18^F-flurpiridaz have approximately 60% retention at peak stress, resulting in higher myocardial activity and image quality for the same injected dose.^34^ These longer half-life tracers typically require lower injected activity and scan-time that can be optimized for the desired image quality. These changes in imaging protocol should only affect the selected value of *ε* in Eq. 2, whereas the weight-dependence of cardiac PET image quality (*β*) is expected to remain the same regardless of these tracer and protocol changes. The present study value of *ε* = 0.1 MBq·kg^−1^ was selected to maintain the same ^82^Rb image quality as our previous clinical standard dosing protocol (9 MBq·kg^−1^) for our historical average patient weight of 90 kg. This value is higher than those reported previously (0.023 to 0.053 MBq·kg^−2^) to standardize ^18^FDG PET image quality, likely due to the ultra-short half-life of ^82^Rb resulting in much lower count-rate and image quality recorded per unit activity (MBq) injected. Exponential dosing for ^13^N-ammonia would likely use a value of *ε* closer to those used in prior ^18^FDG studies, as the typical scan times are close to the isotope half-life of 10 min.

### Study limitations

The effects of exponential versus proportional dosing were evaluated only on stress perfusion image quality, however similar results are expected for perfusion imaging at rest. Only weight-based dosing was investigated in the present study, whereas other measures of patient body habitus such as body mass index, body surface area, chest circumference, etc. could be considered as the patient-specific factor used to prescribe the injected activity. Many of these factors were investigated in the original ^18^FDG study by de Groot which found that patient body weight was the best predictor of changes in image quality,^23^ therefore we followed the same approach and observed similar dosing protocol-dependent results for ^82^Rb PET.

Most of the patients evaluated in this study were in the range of 50 to 120 kg, however many patients at highest risk for CAD may be heavier than 120 kg. The maximum activity of 3700 MBq (100 mCi) available from the ^82^Rb generator^33^ enables exponential dosing in patients up to ≈ 190 kg (420 lbs), but further studies are needed to confirm effectiveness in this obese population, and to evaluate the trend toward improved image quality in the largest patients. The small reduction of injected activity in the exponential- vs proportional-dosing cohort was a by-product of our average cohort weight < 90 kg. Conversely, for patient populations > 90 kg the average injected activity is expected to increase if the same exponential dosing factor is used, i.e., *ε* = 0.1 MBq/kg^2^.

SNR in the LV myocardium could not be measured using the same method as the liver, i.e., SNR_LV_ = LV_MEAN_/LV_SD_ as the values of LV_SD_ were not available in the Corridor-4DM analysis software, but could be the subject of future investigations. The values of LV_SD_ would also be affected by variations in tracer uptake due to CAD, therefore any future studies of SNR_LV_ would be recommended in subjects without CAD to ensure homogeneous tracer uptake.

We did not investigate the effects of exponential dosing on the quantification of myocardial blood flow (MBF). In a previous study, we have shown that PET detector saturation due to dead-time effects can bias the measurements of MBF when the bolus first-pass count-rate exceeds the scanner’s dynamic range.^18^ In centers performing MBF quantification, the injected activity must be kept below some maximum value which maintains accuracy of the bolus first-pass dynamic images, and this may limit the implementation of exponential dosing in larger patients. Saturation bias is PET scanner-specific and can be characterized easily as a function of the dynamic prompt coincidence count-rate.^16,35^ Unfortunately, these values are not saved currently in the reconstructed image DICOM headers by the PET vendor used in this study; this may limit the ability to perform routine quality assurance of MBF accuracy in clinical practice when using the exponential dosing protocol. In these patients there remains a trade-off between standardization of perfusion image quality versus accurate quantification MBF. The study of Moody et al. suggested that BMI-based dosing may be used to lower the incidence of PET saturation compared to proportional weight-based dosing.^36^ Patient BMI (kg/m^2^) is also proportional to weight therefore an exponential function of BMI may help to minimize saturation effects and maintain MBF accuracy while also standardizing ^82^Rb PET image quality.

## New Knowledge Gained

Administration of ^82^Rb activity as a fixed constant dose or in proportion to weight, as recommended in current guidelines, still results in stress PET perfusion image quality that decreases with patient weight. Exponential dosing as a squared function of patient weight (0.1 MBq·kg^−2^) was found to standardize ECG-gated image quality across a wide range of weights, consistent with the goals of high-quality and patient-centered imaging. The proposed protocol can distribute the population dose from the smaller toward the larger patients as needed to maintain image quality, without increasing the average dose.

## Conclusion

^82^Rb PET perfusion image quality is degraded in larger patients when the injected activity is kept at a single constant value. This effect is still observed (but to a lesser degree) when the activity is increased in proportion to patient weight. Administration of ^82^Rb activity as a squared function of patient weight was effective to reduce the weight-dependence of image quality for patients in the range of 50 to 120 kg. This dosing protocol is recommended to standardize MPI quality when feasible within the limits of ^82^Rb generator activity levels. Further studies are needed to evaluate the interaction of exponential dosing and PET scanner dynamic range on the accuracy of MBF quantification, particularly in patients > 120 kg where detector saturation effects are more pronounced.

### Supplementary Information

Below is the link to the electronic supplementary material.

Supplementary file1 (DOCX 1649 KB)

Supplementary file2 (PPTX 513 KB)

Supplementary file3 (MP3 513 KB)

## Abbreviations

CNR: Contrast-to-Noise Ratio
IQS: Image Quality Score
MPI: Myocardial perfusion imaging
PET: Positron emission tomography
^82^Rb: Rubidium-82
SNR: Signal-to-Noise Ratio
SPECT: Single photon emission computed tomography
LV: Left ventricle
