# Supplementary material for: Exponential dosing to standardize myocardial perfusion image quality with rubidium-82 PET
Source: J Nucl Cardiol. 2023 May 31;30(6):2477–89. doi: 10.1007/s12350-023-03303-6 (PMC10682245; doi:10.1007/s12350-023-03303-6)
Supplement: Supplementary file 1 — Supplementary file1 (DOCX 1649 KB) [file 12350_2023_3303_MOESM1_ESM.docx]

Supplemental Materials:

**Exponential Dosing to Standardize Myocardial Perfusion Image Quality with Rubidium-82 PET**

Anahita Tavoosi, Ritika Khetarpal, R. Glenn Wells, Rob S.B. Beanlands, Robert A. deKemp

**Table S1**. Operator reproducibility of image quality measurements

| **Dosing Cohort** | **IQS _HEART_** | **CNR _HEART_** | **SNR _BLOOD_** |
| --- | --- | --- | --- |
| Static Proportional | - | 4 ± 37% | 5 ± 35% |
| Gated Proportional | −2 ± 14% | 2 ± 34% | 1 ± 32% |
| Static Exponential | - | −3 ± 34% | −1 ± 32% |
| Gated Exponential | −2 ± 15% | 0 ± 32% | 0 ± 27% |

Values are mean difference ± standard deviation between operators

No significant differences versus zero

IQS Imaging quality score, CNR Contrast-to-noise ratio, SNR Signal-to-noise ratio

**
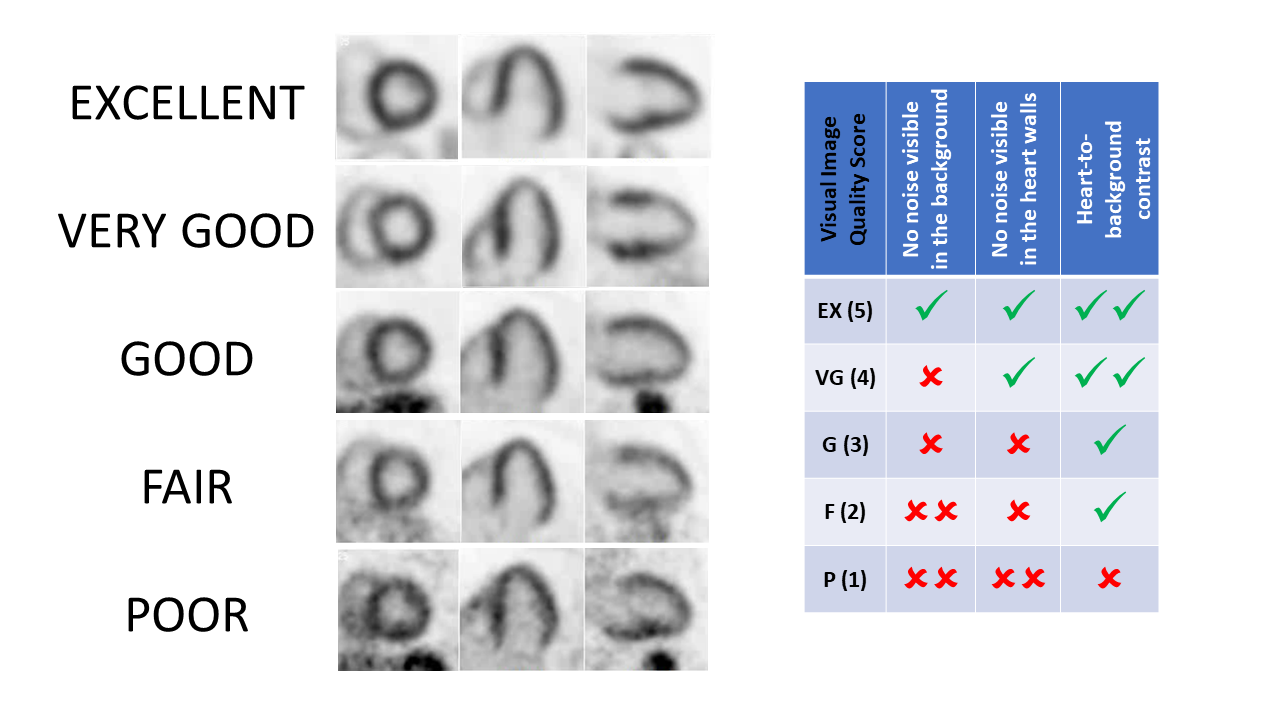
**

**Figure S1.** Training images (left) and criteria (right) used to guide visual image scoring by the physicians.

| **A**  **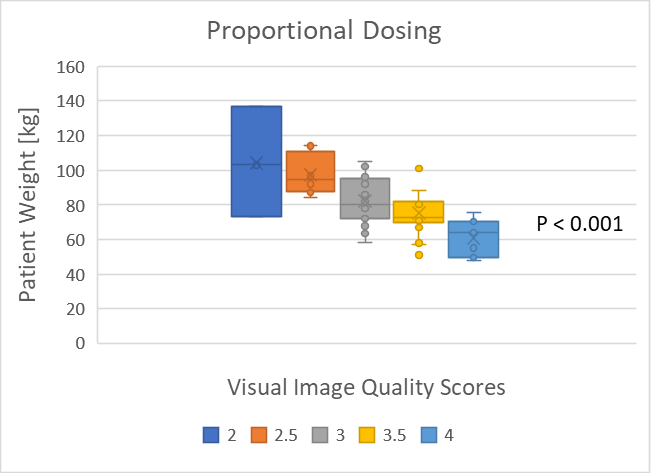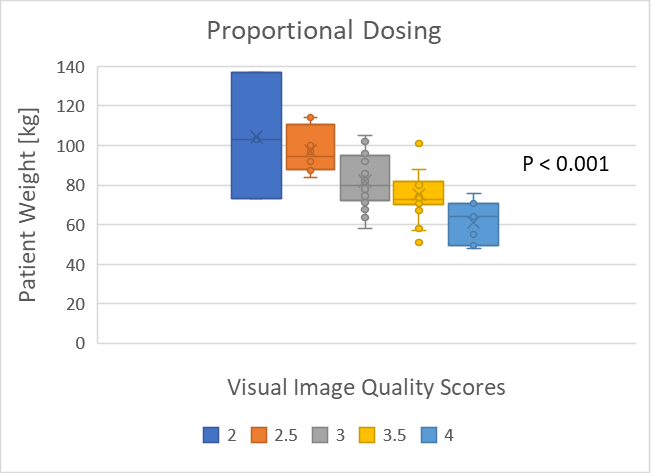** | **B**  **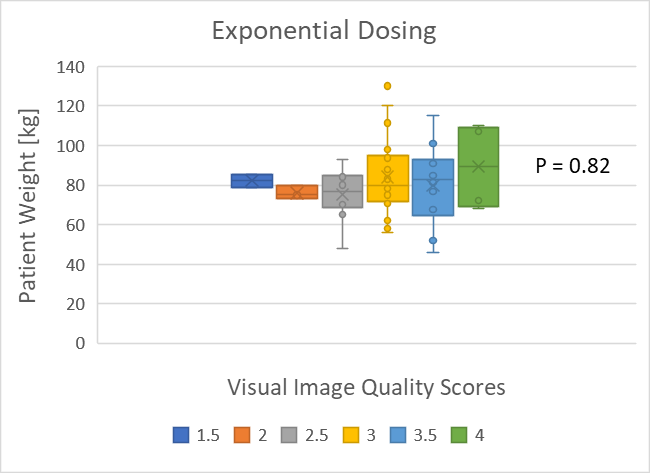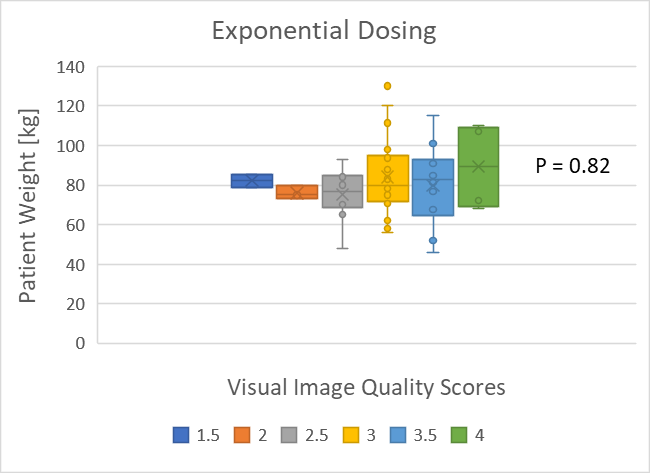** |
| --- | --- |

**Figure S2**. Box-plots of patient weight according to visual image quality score (IQS) in the proportional (A) and exponential (B) dosing groups. There was a highly significant effect of increasing weight in patients with lower IQS in the proportion dosing group (P < 0.001), whereas there was no such effect observed in the exponential dosing group (P = 0.82) using Kruskal-Wallis tests.

| **A**  51kg proportional dosing: ungated-CNR **200**  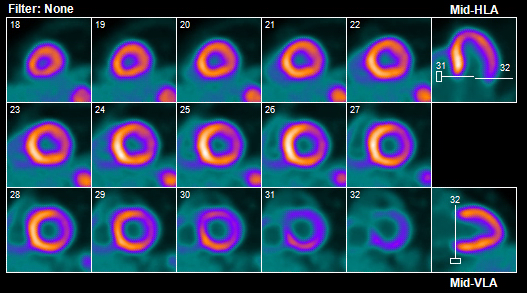 | **B**  114kg proportional dosing: ungated-CNR **45**  **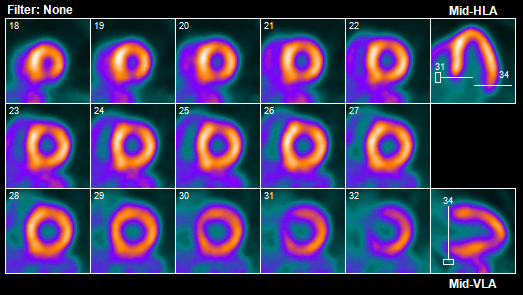** |
| --- | --- |
| **C**  52kg exponential dosing: ungated-CNR **120**  **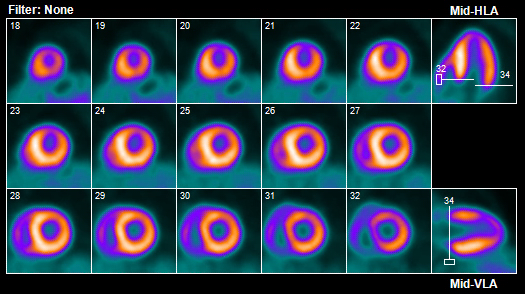** | **D**  131kg exponential dosing: ungated-CNR **70**  **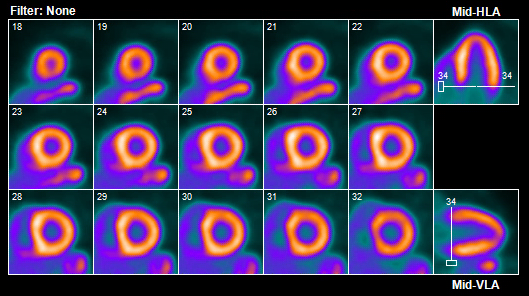** |

**Figure S3**. ^82^Rb PET static (ungated) images acquired with proportional (A,B) and exponential (C,D) dosing for the same patients shown in Figure 6. Proportional dosing resulted in lower image quality in the large (B) vs small (A) patient (CNR = 45 vs 200). With exponential dosing the image quality was more similar between the large (D) and small (C) patient (CNR = 70 vs 120) and much improved vs the large patient with proportional dosing (B).

| **A**  **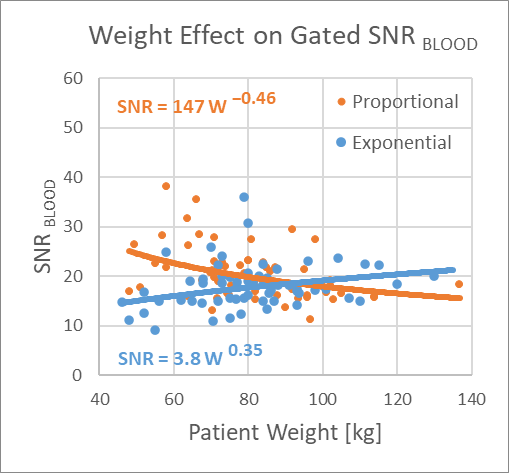** | **B**  **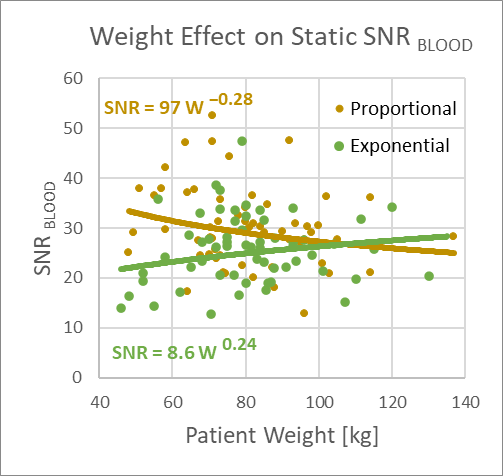** | **C**  **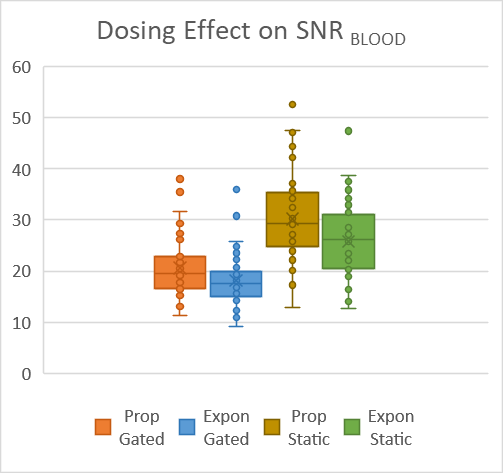** |
| --- | --- | --- |

**Figure S4**. ^82^Rb PET signal-to-noise ratio (SNR_BLOOD_) ***decreases*** with increasing patient body weight in the proportional dosing cohort (A) and tended to ***increase*** in the exponential dosing cohort (B). Box-plots of the SNR_BLOOD_ (C) show the summary effects of dosing method on the patient groups as a whole.

Lines of best-fit are ***SNR* ∝** ***Weight* ^β^**

| **A**  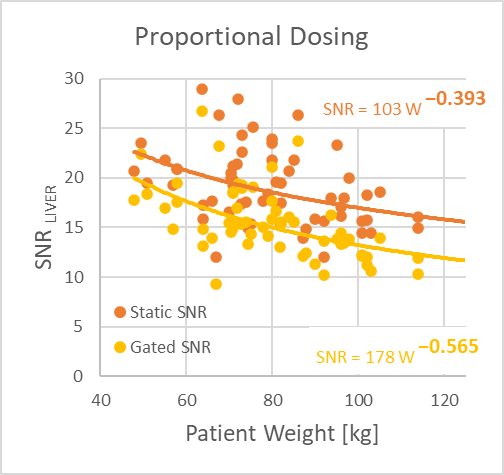 | **B**  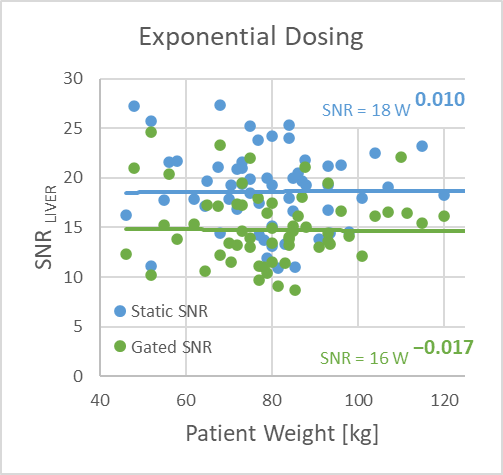 | **C**  **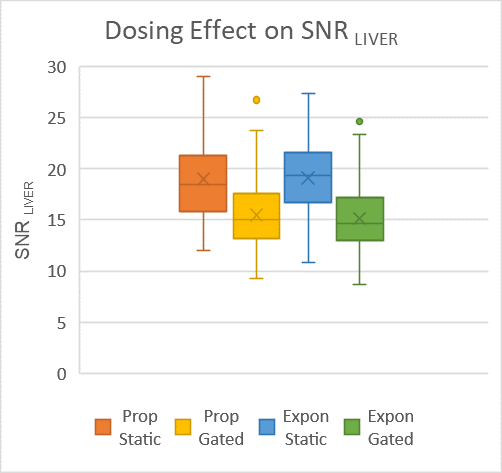** |
| --- | --- | --- |

**Figure S5**. ^82^Rb PET liver signal-to-noise ratio (SNR_LIVER_) ***decreases*** with increasing patient body weight (W) in the proportional dosing cohort (A) but not in the exponential dosing cohort (B). Box-plots of the SNR_LIVER_ (C) show the summary effects of dosing method on the patient groups as a whole.

Lines of best-fit are ***SNR* ∝** ***Weight* ^β^**
