# Supplementary material for: Exponential dosing to standardize myocardial perfusion image quality with rubidium-82 PET
Source: J Nucl Cardiol. 2023 May 31;30(6):2477–89. doi: 10.1007/s12350-023-03303-6 (PMC10682245; doi:10.1007/s12350-023-03303-6)
Supplement: Supplementary file 2 — Supplementary file2 (PPTX 513 KB) [file 12350_2023_3303_MOESM2_ESM.pptx]

## Slide 1
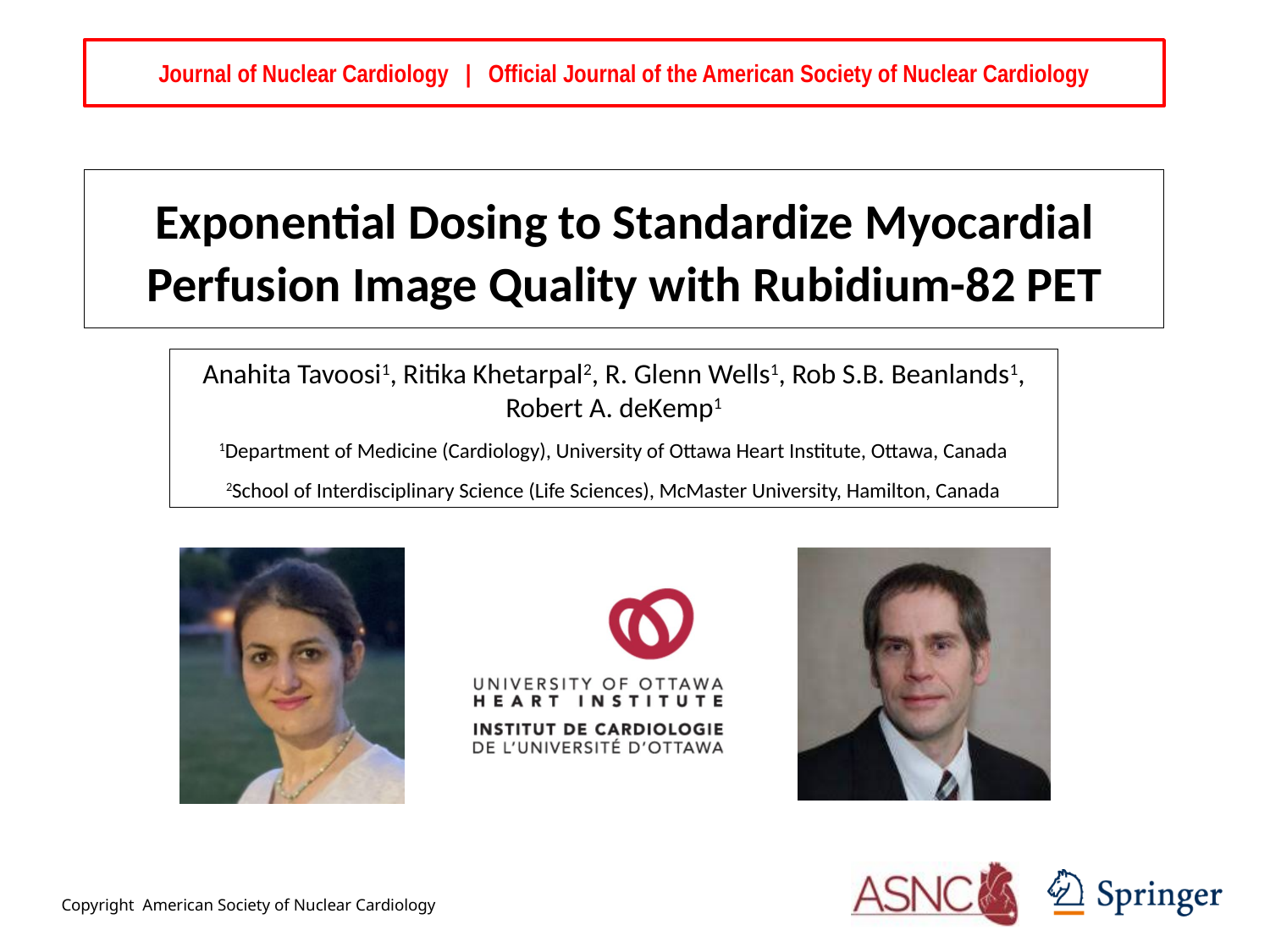

Journal of Nuclear Cardiology | Official Journal of the American Society of Nuclear Cardiology
# Exponential Dosing to Standardize Myocardial Perfusion Image Quality with Rubidium-82 PET
Anahita Tavoosi1, Ritika Khetarpal2, R. Glenn Wells1, Rob S.B. Beanlands1, Robert A. deKemp1
1Department of Medicine (Cardiology), University of Ottawa Heart Institute, Ottawa, Canada
2School of Interdisciplinary Science (Life Sciences), McMaster University, Hamilton, Canada
Copyright American Society of Nuclear Cardiology

## Slide 2
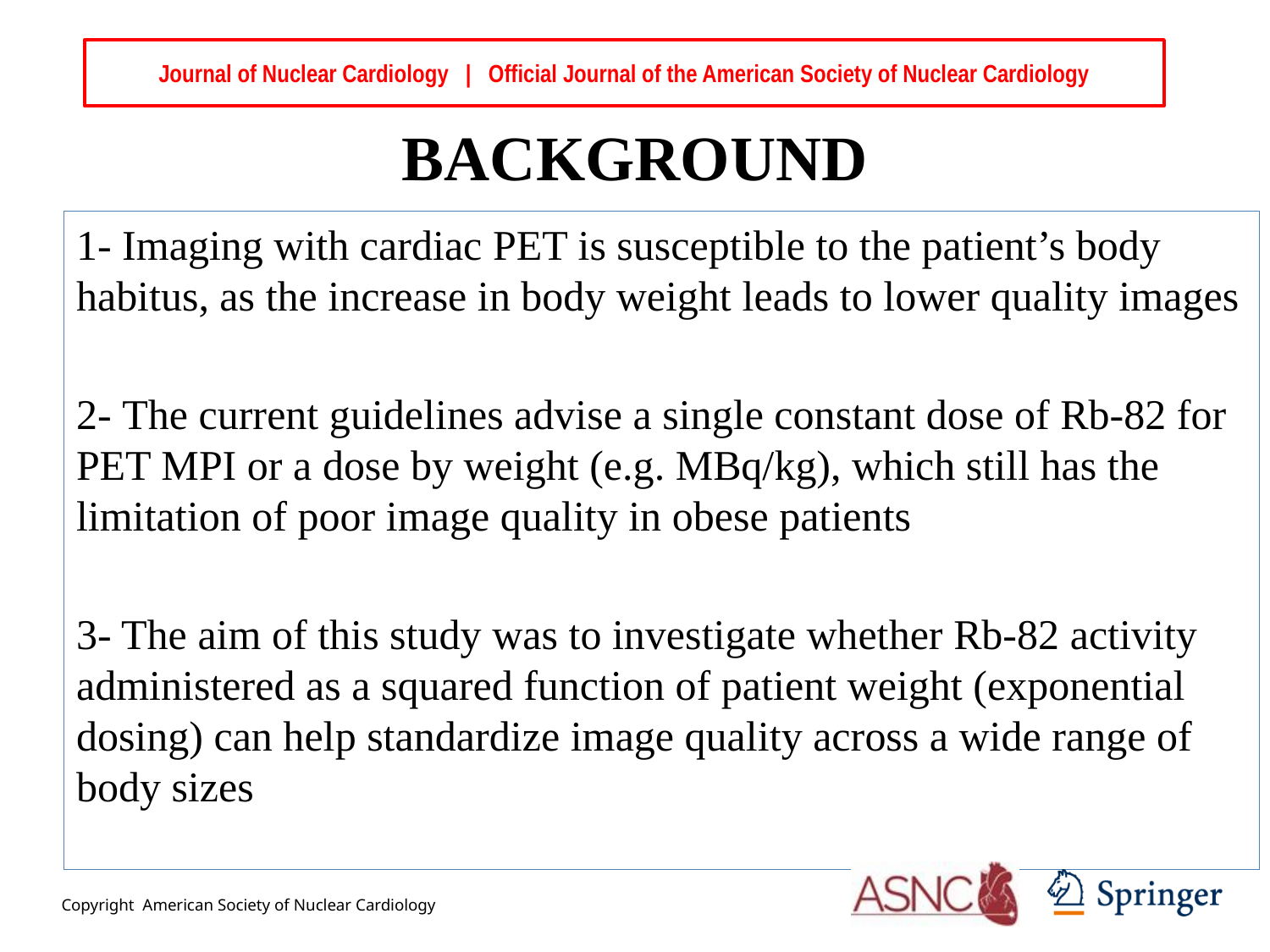

Journal of Nuclear Cardiology | Official Journal of the American Society of Nuclear Cardiology
# BACKGROUND
1- Imaging with cardiac PET is susceptible to the patient’s body habitus, as the increase in body weight leads to lower quality images
2- The current guidelines advise a single constant dose of Rb-82 for PET MPI or a dose by weight (e.g. MBq/kg), which still has the limitation of poor image quality in obese patients
3- The aim of this study was to investigate whether Rb-82 activity administered as a squared function of patient weight (exponential dosing) can help standardize image quality across a wide range of body sizes
Copyright American Society of Nuclear Cardiology

## Slide 3
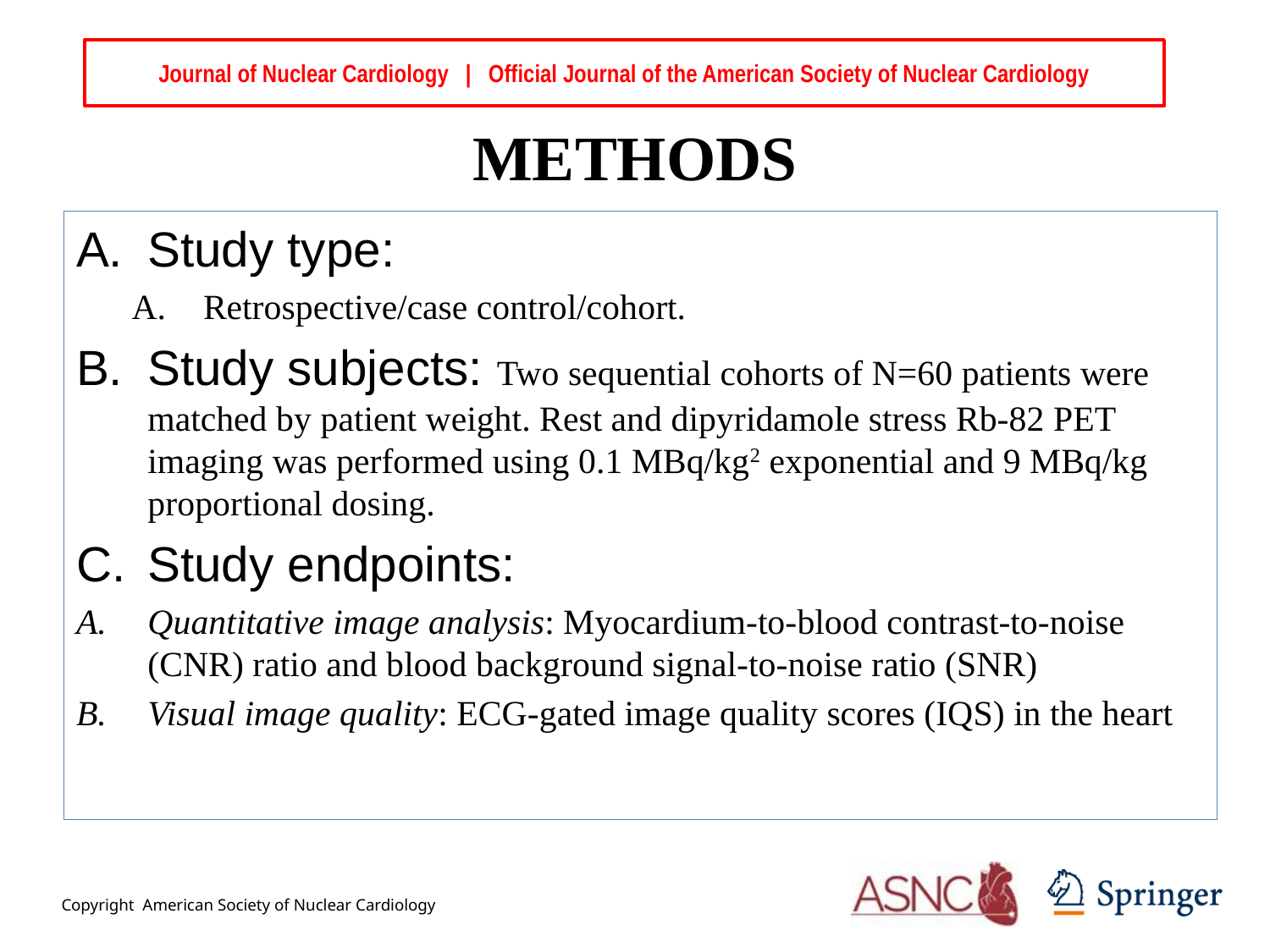

Journal of Nuclear Cardiology | Official Journal of the American Society of Nuclear Cardiology
# METHODS
Study type:
Retrospective/case control/cohort.
Study subjects: Two sequential cohorts of N=60 patients were matched by patient weight. Rest and dipyridamole stress Rb-82 PET imaging was performed using 0.1 MBq/kg2 exponential and 9 MBq/kg proportional dosing.
Study endpoints:
Quantitative image analysis: Myocardium-to-blood contrast-to-noise (CNR) ratio and blood background signal-to-noise ratio (SNR)
Visual image quality: ECG-gated image quality scores (IQS) in the heart
Copyright American Society of Nuclear Cardiology

## Slide 4
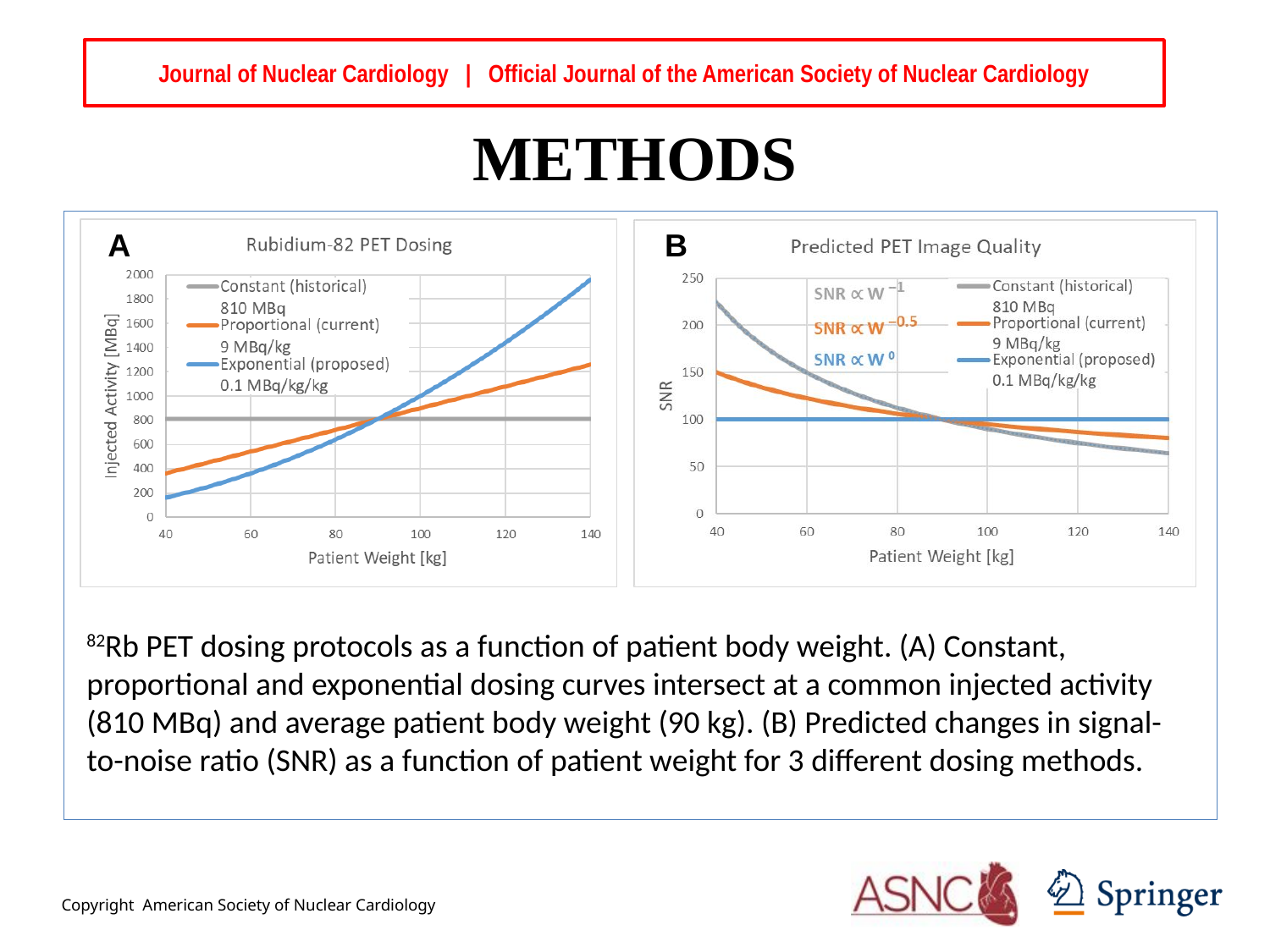

Journal of Nuclear Cardiology | Official Journal of the American Society of Nuclear Cardiology
# METHODS
A
B
82Rb PET dosing protocols as a function of patient body weight. (A) Constant, proportional and exponential dosing curves intersect at a common injected activity (810 MBq) and average patient body weight (90 kg). (B) Predicted changes in signal-to-noise ratio (SNR) as a function of patient weight for 3 different dosing methods.
Copyright American Society of Nuclear Cardiology

## Slide 5
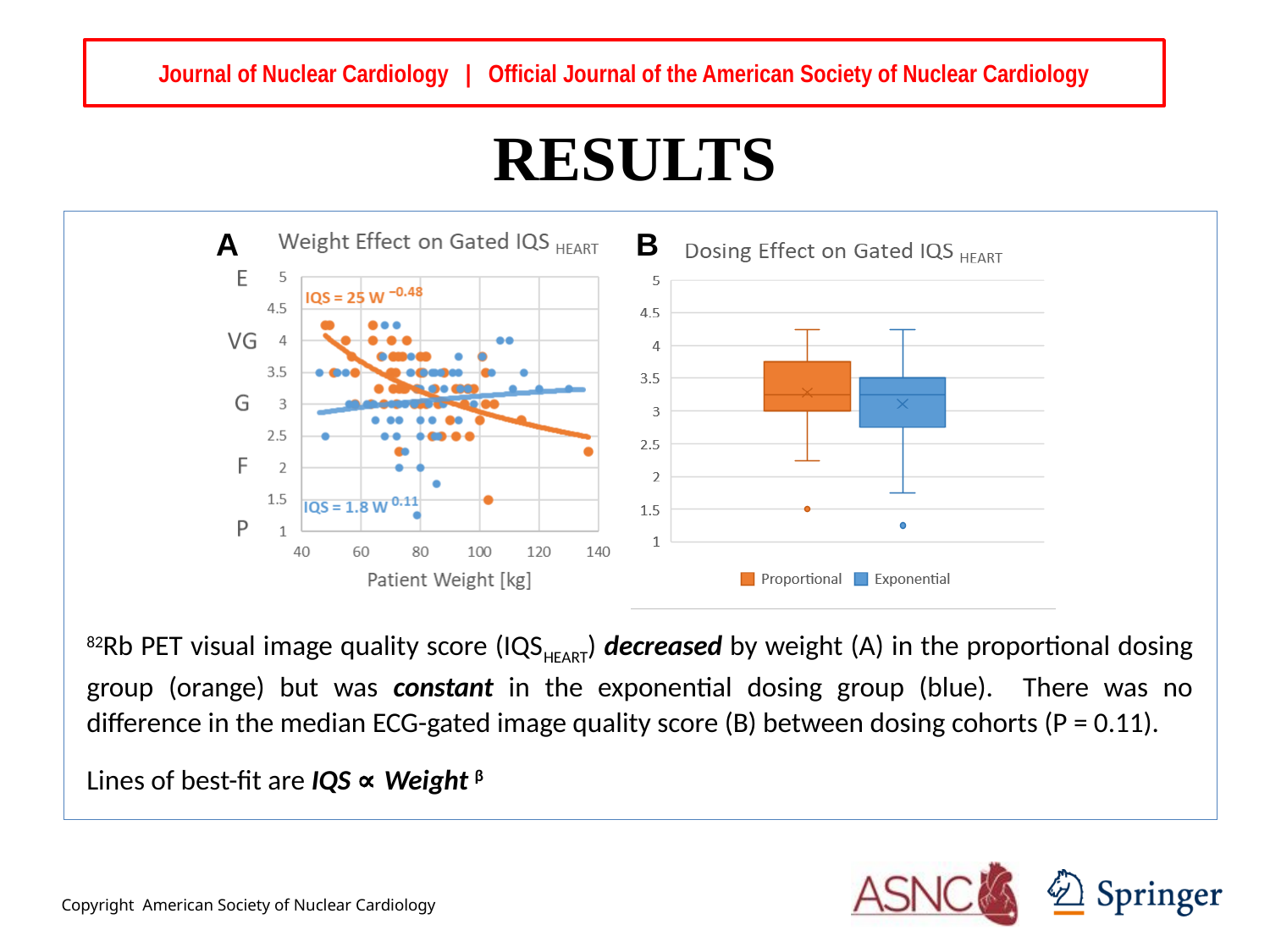

Journal of Nuclear Cardiology | Official Journal of the American Society of Nuclear Cardiology
# RESULTS
A
B
82Rb PET visual image quality score (IQSHEART) decreased by weight (A) in the proportional dosing group (orange) but was constant in the exponential dosing group (blue). There was no difference in the median ECG-gated image quality score (B) between dosing cohorts (P = 0.11).
Lines of best-fit are IQS ∝ Weight β
Copyright American Society of Nuclear Cardiology

## Slide 6
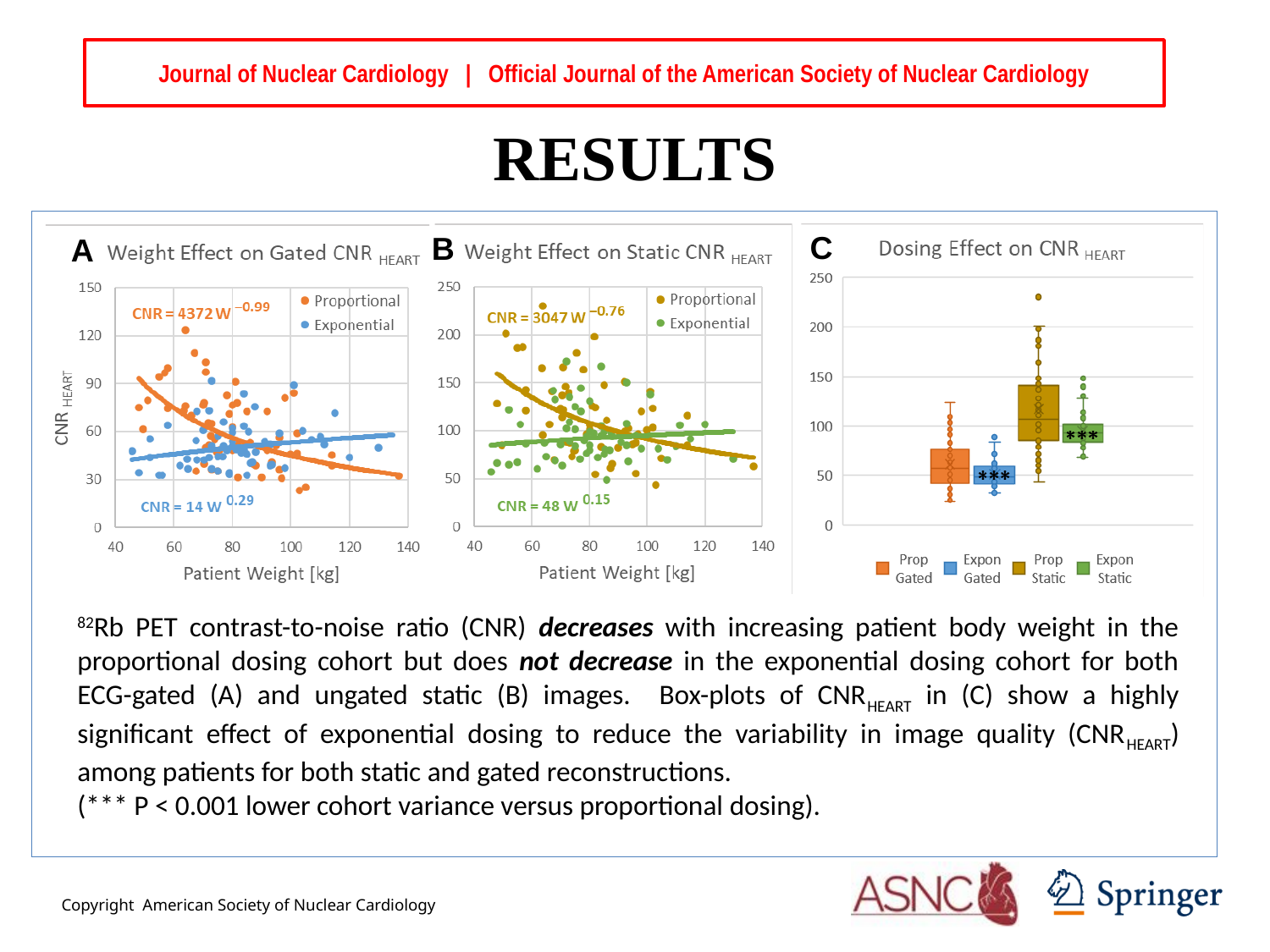

Journal of Nuclear Cardiology | Official Journal of the American Society of Nuclear Cardiology
# RESULTS
C
B
A
82Rb PET contrast-to-noise ratio (CNR) decreases with increasing patient body weight in the proportional dosing cohort but does not decrease in the exponential dosing cohort for both ECG-gated (A) and ungated static (B) images. Box-plots of CNRHEART in (C) show a highly significant effect of exponential dosing to reduce the variability in image quality (CNRHEART) among patients for both static and gated reconstructions.
(*** P < 0.001 lower cohort variance versus proportional dosing).
Copyright American Society of Nuclear Cardiology

## Slide 7
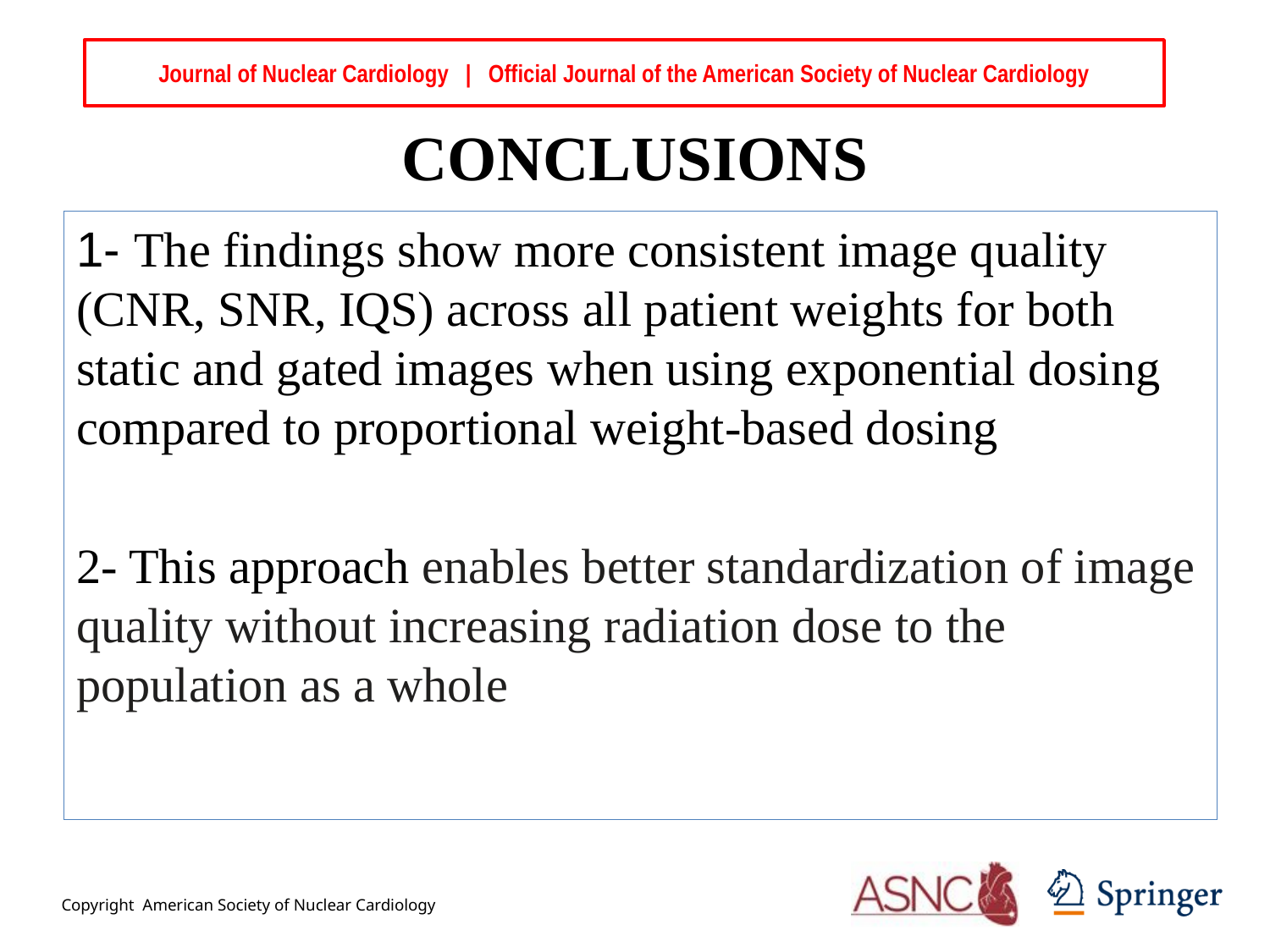

Journal of Nuclear Cardiology | Official Journal of the American Society of Nuclear Cardiology
# CONCLUSIONS
1- The findings show more consistent image quality (CNR, SNR, IQS) across all patient weights for both static and gated images when using exponential dosing compared to proportional weight-based dosing
2- This approach enables better standardization of image quality without increasing radiation dose to the population as a whole
Copyright American Society of Nuclear Cardiology
